# A TAP1 null mutation leads to an enlarged olfactory bulb and supernumerary, ectopic olfactory glomeruli

**DOI:** 10.1098/rsob.130044

**Published:** 2013-05

**Authors:** Ernesto Salcedo, Nicole M. Cruz, Xuan Ly, Beth A. Welander, Kyle Hanson, Eugene Kronberg, Diego Restrepo

**Affiliations:** Cell and Developmental Biology, Rocky Mountain Taste and Smell Center, University of Colorado School of Medicine, Aurora, CO, USA

**Keywords:** olfaction, main histocompatibility complex I, P2 glomerulus, main olfactory bulb, major histocompatibility class I molecules

## Abstract

Major histocompatibility class I (MHCI) molecules are well known for their immunological role in mediating tissue graft rejection. Recently, these molecules were discovered to be expressed in distinct neuronal subclasses, dispelling the long-held tenet that the uninjured brain is immune-privileged. Here, we show that MHCI molecules are expressed in the main olfactory bulb (MOB) of adult animals. Furthermore, we find that mice with diminished levels of MHCI expression have enlarged MOBs containing an increased number of small, morphologically abnormal and ectopically located P2 glomeruli. These findings suggest that MHCI molecules may play an important role in the proper formation of glomeruli in the bulb.

## 2. Introduction

The discovery that major histocompatibility class I (MHCI) molecules are expressed in healthy neurons uncovered an unexpected role for these molecules in synaptic remodelling and plasticity in several different neuronal systems [1–11]. For example, in mice, severely deficient in MHCI expression, retinal afferents fail to properly segregate into eye-specific layers of the lateral geniculate nucleus (LGN) despite the presence of normal retinal activity [2]. MHCI molecules, their putative receptors and transporter associated with antigen processing protein (TAP1), which is required for the presentation of a MHCI molecules on cell surfaces, have all been detected in the olfactory system [12–15]. However, the role these molecules play in the neuronal refinement and plasticity of olfactory circuits as seen in other systems remains to be determined.

The glomerular layer of the MOB offers a powerful and highly accessible model system to investigate the role of MHCI molecules in the proper formation and maintenance of olfactory circuits. Here, axons from millions of olfactory sensory neurons (OSNs) synapse onto tens of thousands of second-order neurons in thousands of spherical neuropils called glomeruli. Each glomerulus receives axons only from OSNs that express the same odorant receptor (OR), and thus shares the same molecular receptive field as its pool of incoming OSNs. OSNs expressing the same OR reliably target the same pair of symmetrically opposed glomeruli found in positionally consistent locations on the surface of the bulb [16,17]. This positional consistency results in a reproducible topographical representation of olfactory activity on the surface of the MOB that is known as an odour map [16,18–21]. While many different molecular determinants have been implicated in the formation of the odour map, the mechanisms by which OSN axons can target individual glomeruli (out of a potential field of approx. 1800) with such precision remains to be fully understood [20,22].

Much of our current understanding of glomerular formation and refinement has benefitted from the characterization of a handful of genetically labelled glomeruli [20,22]. For example, the P2 glomerulus has been extensively characterized using transgenic animals in which the P2 OR was coexpressed with an axonal fusion marker such as tau-lacZ (TLZ) or tau-green fluorescent protein (tGFP) [23]. These studies have shown that during early development, P2 OSN axons target multiple ectopic glomeruli or erroneously enter into the outer plexiform layer of the MOB [24,25]. As a result, newborn mice typically exhibit multiple extra P2 glomeruli [26]. The aberrant targeting of these OSN axons is subsequently refined during maturation of the animal through an activity-dependent process that involves fasciculation and segregation of axon termini [26–29]. By adulthood, supernumerary glomeruli are pruned down to a pair of symmetrically opposed glomeruli in a process that remains poorly understood. This process appears susceptible to environmental factors as a variable number of supernumerary P2 glomeruli in the adult MOB has been reported in different laboratories [17,23,30]. Intriguingly, a recent study has shown MHCI molecules to be expressed in the regions of the olfactory bulb and during developmental time-points known to be associated with P2 glomerular segregation [14].

In this study, we examined mice that lacked the expression of TAP1 [4,31]. In these mice, we find a severe reduction in the expression of MHCI molecules in the MOB. Interestingly, when we crossed the TAP^−/−^ animals to mice that coexpress the P2 OR with TLZ (P2iTLZ), we find an increased number of supernumerary P2 glomeruli located in ectopic positions on the surface of abnormally long MOBs. Our results indicate that abolishing TAP expression affects the refinement of a specific subpopulation of OSN axons into normal glomeruli and that MHCI molecules may play a role in the segregation of OSN axons into OB glomeruli.

## 3. Results

### 3.1. Major histocompatibility class I antibody labels the main olfactory bulb

**Figure 1.**
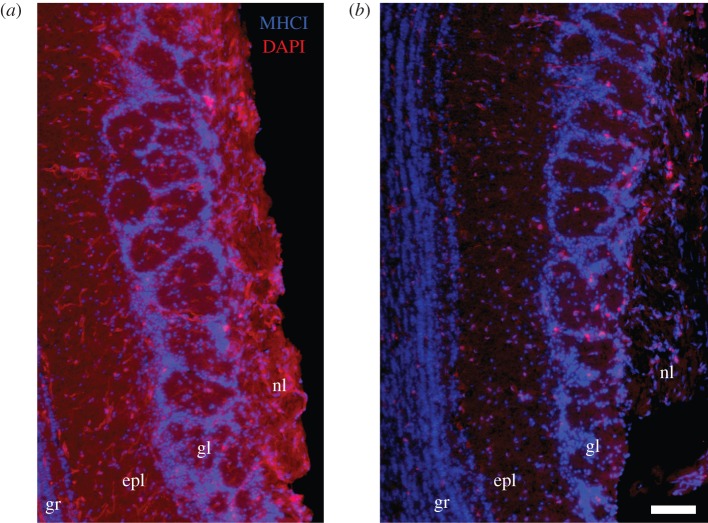
Immunofluorescence labelling for MHCI (red) molecules is greatly reduced in the main olfactory bulb of TAP^−/−^ animals. White scale bar in (*b*) indicates 100 µm (both micrographs to same scale). nl, nerve layer; gl, glomerular layer; epl, external plexiform layer; gr, granule cell layer. Nuclei are labelled with DAPI (blue). MHCI is labelled in red. (*a*) Sample MOB tissue from a C57Bl/6 animal. (*b*) Comparable MOB tissue from a TAP^−/−^ animal.

To determine the effect that TAP^−/−^ mutation has on the expression of MHCI molecules in the MOB, we used a pan-specific MHCI antibody to immunolabel olfactory tissue from C57Bl/6 and TAP^−/−^ adult animals. We detected a diffuse immunostaining pattern for MHCI in the C57Bl/6 animals throughout the laminar layers of the olfactory bulb, including the nerve layer, the glomerular layer, the external plexiform layer, the mitral cell and granule cell layers (figure 1*a*). We also found widespread labelling of the vasculature throughout the tissue and heavy labelling around some periglomerular cells. By contrast, we found a greatly reduced level of expression of MHCI in the TAP^−/−^ animals throughout the bulb (figure 1*b*) that was above the level of the no-primary control, but not completely eliminated.

To further characterize the expression pattern for MHCI, we co-labelled olfactory bulb tissue from C57Bl/6 control animals with the pan-specific MHCI antibody and known immunomarkers for different cell types typically found in the MOB, including those for olfactory sensory neuronal axons (olfactory marker protein, OMP), astrocytes (glial fibrillary acidic protein, GFAP), immature olfactory neurons (growth-associated protein 43, GP-43) and ensheathing cells (p75 low-affinity nerve growth factor receptor). Owing to the diffuse nature of the MHCI staining pattern, we were unable to confirm specific co-localization of these olfactory markers with the pan-specific MHCI immunomarker (data not shown). Nevertheless, the staining pattern seen in figure 1 clearly shows that MHCI molecules can be found in the regions of the MOB, such as the nerve and glomerular layers, that are associated with the fasciculation and segregation of axon termini into glomeruli.

### 3.2. TAP^−/−^ bulbs contain morphologically abnormal P2 glomeruli

**Figure 2.**
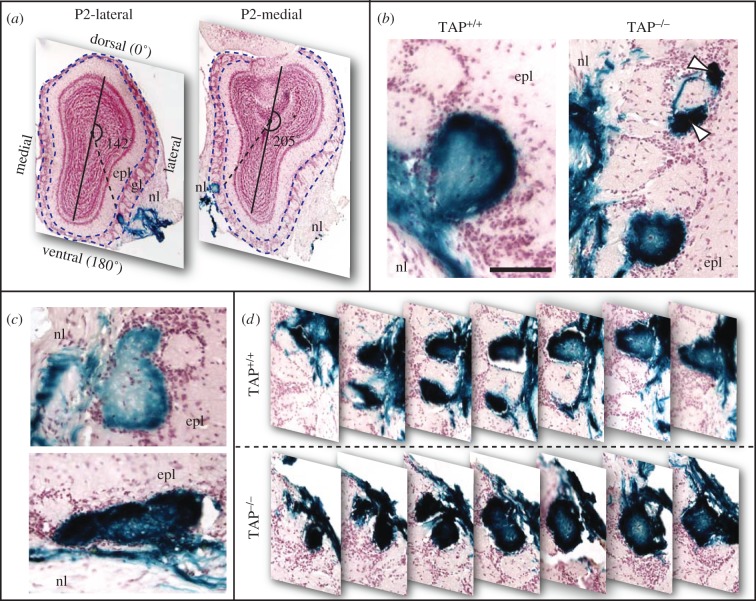
X-Gal-labelled P2 glomeruli in TAP^+/+^ and TAP^−/−^ mice. High-magnification images of cryosections from the main olfactory bulb. These sections were cut parallel to the long axis of the bulb. The external plexiform layer is labelled epl. The olfactory nerve layer is labelled nl. Axons from P2 olfactory sensory neurons are labelled with the blue X-gal dye. (*a*) Two representative cryosections from a P2iTLZ animal with labelled P2 glomeruli in the ventrolateral and ventromedial domains of each olfactory bulb. The black, vertical line extending from dorsal to ventral mitral cell layer is the dorsoventral axis, as described in §5. The dashed blue lines outline the glomerular layer (gl) in each cryosection. As is typical for P2iTLZ animals, the left, more rostral image contains a ventral P2-lateral glomerulus at 150°, whereas the right, more caudal image contains a ventral P2-medial glomerulus at 205°. (*b*) A singlet P2-medial glomerulus in a TAP^+/+^ animal. Left (TAP^+/+^): typical singlet glomerular cross section from a TAP^+/+^ animal. The scale bar indicates 100 µm. Right (TAP^−/−^): a set of triplet P2-medial glomeruli in a TAP^−/−^ animal. Although these glomeruli have a normal morphology, the arrow heads in the right panel point to two exceptionally small glomeruli. (*c*) Two distinct, abnormally fused P2 glomeruli seen in TAP^−/−^ animals. (*d*) Serial montage of glomerular cross sections from a TAP^+/+^ doublet (top panel) with one intervening, unlabelled glomerulus and a fused TAP^−/−^ doublet (bottom panel) with zero intervening glomeruli.

To investigate a role for MHCI molecules in the formation and maintenance of glomerular architecture, we generated mice that were homozygous for both the P2iTLZ transgene and TAP^−/−^. On gross inspection, we found no evident anatomical defects in the MOE or MOB in TAP^−/−^ animals. To visualize the somas and axons from OSNs that express the P2 OR, we used the X-gal reaction to stain tissue from TAP^−/−^ animals and TAP^+/+^ mice that contained the P2iTLZ transgene (figure 2). We found the presence of X-gal dye appropriately restricted to the outer nerve and glomerular layers of the MOB in TAP^−/−^ mice (figure 2*a*), consistent with the expression of P2iTLZ in a subset of OSN axons and in line with previous reports of P2iTLZ expression in the bulb [17,23]. Interestingly, we noted a number of morphologically abnormal P2 glomeruli in the TAP^−/−^ bulbs (figure 2*b–d*). Some glomeruli were exceptionally small (figure 2*b*), whereas others closely resembled the fused P2 glomeruli reported during early postnatal development (figure 2*c*) [24]. These fused glomeruli appear to be joined at one or more tissue cross sections, but ultimately bifurcate into independent units in subsequent cross sections (figure 2*d*). By contrast, we did not find such similarly fused glomeruli in the TAP^+/+^ bulbs from comparable littermates.

### 3.3. Dramatic increase in the number of P2 glomeruli in mice deficient for membrane expression of MHCI

**Figure 3.**
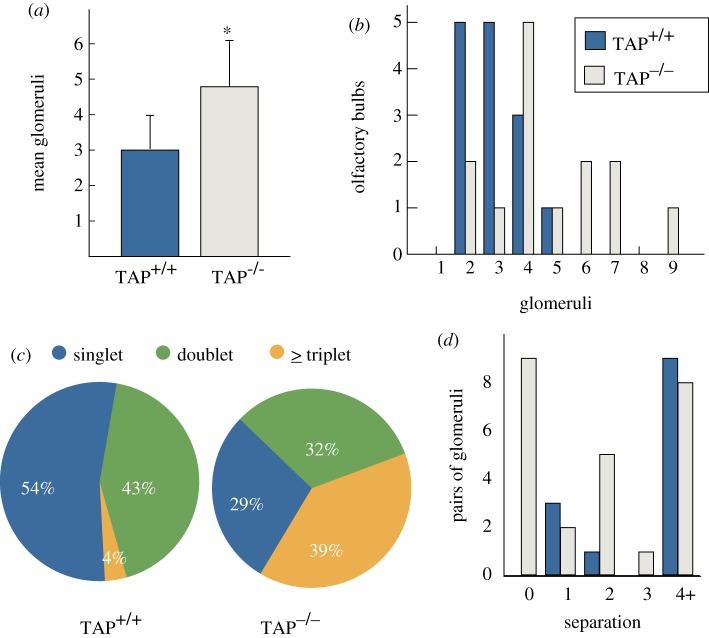
TAP^−/−^ animals have a significant increase in the number of supernumerary P2 glomeruli. (*a*) The average number of TAP^−/−^ P2 glomeruli, 4.8 ± 1.4 (±s.e.), is significantly larger than the number of TAP^+/+^ P2 glomeruli, 3 ± 0.98 (mixed-effects analysis ANOVA, *p* = 0.037). (*b*) TAP^−/−^ animals have a significantly higher incidence of bulbs with four or more P2 glomeruli per bulb. (*c*) TAP^−/−^ bulbs have a higher incidence of multiplet glomeruli in a given lateral (P2-lateral) or medial (P2-medial) domain. Singlet: one glomerulus in a given lateral or medial domain of a bulb. Doublet: two glomeruli per domain. Triplet or greater: three or more glomeruli per domain. (*d*) The number intervening glomeruli between all pairs of duplicate P2 glomeruli. TAP^+/+^ animals had a total of 13 pairs of P2 glomeruli (36%) that qualified as duplicate glomeruli, whereas TAP^−/−^ animals had 25 pairs. Nine TAP^−/−^ glomeruli had the fused morphology (0 intervening glomeruli), whereas no TAP^+/+^ glomerulus had a similar morphology.

As shown in figure 3*a*, TAP^+/+^ bulbs had an average of 3 ± 0.98 (±s.e.) glomeruli (a total of 42 glomeruli in 14 bulbs, *n* = 7 animals), ranging from two to five glomeruli per bulb. These numbers are consistent with previous studies that uniformly report finding on average two symmetrically opposed glomeruli (P2-lateral and P2-medial) per bulb in adult mice [17,23,24,30,32]. TAP^−/−^ animals, by comparison, had an average of 4.8 ± 1.4 P2 glomeruli per bulb (67 glomeruli in 14 bulbs, *n* = 7 animals): a significant increase in the total number of glomeruli per bulb (mixed-effects analysis ANOVA, *p* = 0.0373). Furthermore, whereas only one TAP^+/+^ bulb had more than four P2 glomeruli, nearly 43 per cent of the TAP^−/−^ bulbs had five or more P2 glomeruli, and one bulb had a total of nine P2 glomeruli (figure 3*b*). These results indicate that TAP^−/−^ animals had a dramatic increase in the overall number of P2 glomeruli per bulb.

To determine whether this increase in the total number of glomeruli represented a disproportionate increase in the number of P2-lateral or P2-medial glomeruli, we binned the number of P2 glomeruli per lateral or medial domain. Previous reports indicate that each domain should contain at least one P2 glomerulus and up to two glomeruli [17,23,24,32]. We found TAP^+/+^ animals had an average of 1.64 ± 0.5 and 1.36 ± 0.63 (±s.d.) P2-lateral and P2-medial glomeruli, respectively. Consistent with the increased overall number of P2 glomeruli per bulb, TAP^−/−^ animals had an average of 2.50 ± 1.51 and 2.29 ± 1.14 P2-lateral and P2-medial glomeruli, respectively. We found no statistical difference in the number of glomeruli per domain within strains in either the TAP^+/+^ (mixed-effects ANOVA, *p* = 0.10) or TAP^−/−^ (mixed-effects ANOVA, *p* = 0.66) animals. Thus, the number of P2 glomeruli, regardless of total count, appears to be evenly distributed between the lateral and medial domains of both groups of animals, consistent with previous results. Nevertheless, TAP^−/−^ animals consistently contained an increased number of P2 glomeruli per domain when compared with their littermate controls. As shown in figure 3*c*, more than half (54%) of the TAP^+/+^ lateral or medial domains contained one P2 glomerulus, 43 per cent contained two glomeruli, and only one single domain (4%) contained three glomeruli. By comparison, less than one-third of TAP^−/−^ domains contained a single glomerulus (chi-squared test, *p* = 0.06), nearly one-third contained two glomeruli (chi-squared test, *p* = 0.41), and 39 per cent had three or more glomeruli (chi-squared test, *p* = 0.0011). These data indicate that TAP^−/−^ animals had a far greater incidence of multiple P2 glomeruli in either the lateroventral or medioventral domains of each olfactory bulb when compared with their control littermates.

To quantify the incidence of fused glomeruli, we counted the number of intervening glomeruli at the closest cross-sectional juncture between all multiplet pairs of P2 glomeruli. This metric allows us to differentiate fused glomeruli from the proximal multiplet pairs that are seen in TAP^+/+^ bulbs. For example, we counted one intervening glomerulus between the multiplet pair shown in figure 2*b* and no intervening glomeruli in the triplet shown in figure 2*d*. As seen in figure 3*d*, the large majority of TAP^+/+^ P2 glomeruli (69%) had a minimum separation of four or more intervening glomeruli between multiplet pairs. By comparison, only 32 per cent of the TAP^−/−^ multiplet pairs had a similar separation (chi-squared test, *p* = 0.028). Furthermore, no pair of TAP^+/+^ P2 glomeruli had the abnormally fused morphology, whereas nine pairs of TAP^−/−^ P2 glomeruli did (36%, chi-squared test, *p* = 0.013). The fact that these morphologically abnormal glomeruli resemble P2 glomeruli seen during early development suggests that TAP^−/−^ animals may have a disruption in the fasciculation process that coalesces OSN axons into single glomeruli.

### 3.4. TAP^−/−^ mice have more abnormally small P2 glomeruli

**Figure 4.**
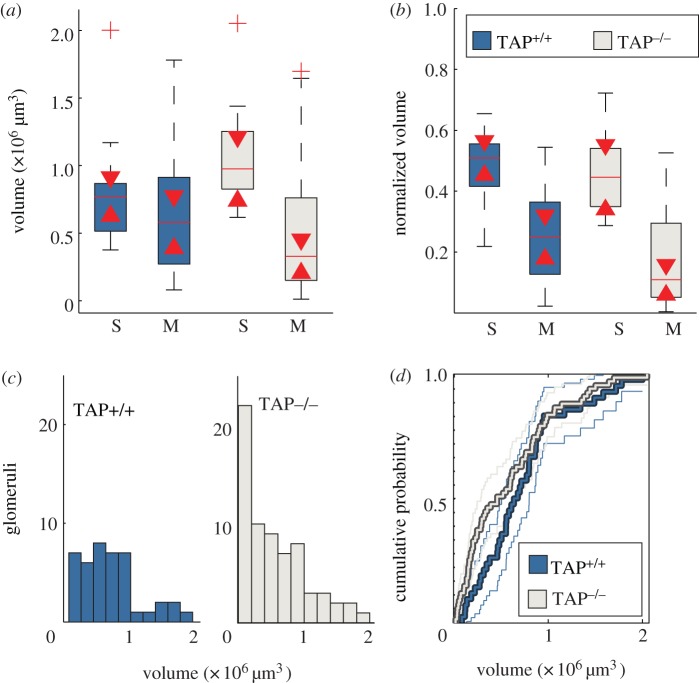
TAP^−/−^ animals have a significant increase in small P2 glomeruli. (*a*) Box plots of singlet (S) or multiplet (M) P2 glomerular volumes in TAP^−/−^ (beige) or TAP^+/+^ (blue) bulbs. Triangle centres indicate 95% confidence interval. The singlet TAP^−/−^ glomeruli triangle interval does not overlap with triangle interval of TAP^−/−^ multiplets, indicating that singlet TAP^−/−^ glomeruli are significantly larger than multiplet TAP^−/−^ glomeruli at the 95% confidence level. (*b*) Glomerular volume normalized to the total glomerular volume of a given bulb. Median singlet glomerular normalized volume is 0.51 ± 0.03 for TAP^+/+^ glomeruli and 0.44 ± 0.05 for TAP^−/−^ glomeruli. Median multiplet normalized volume is 0.25 ± 0.05 for TAP^+/+^ glomeruli and 0.11 ± 0.02 for TAP^−/−^ glomeruli. Triangle ranges do not overlap for multiplet glomeruli, indicating that TAP^−/−^ multiplet glomeruli are significantly smaller than TAP^+/+^ glomeruli. (*c*) Histogram of all glomerular volumes for TAP^+/+^ (blue) or TAP^−/^ (beige) animals highlights the large incidence of P2 TAP^−/−^ glomeruli with volumes calculated to be below 0.4 × 10^6^ μm^3^. (*d*) Cumulative probability of glomerular volumes, indicating significant difference in the volumes of TAP^+/+^ and TAP^−/−^ glomeruli between 0 and 0.8 × 10^6^ μm^3^.

We have previously shown that a given singlet P2 glomerulus (one glomerulus in a given medial or lateral domain) has a larger volume on average than the volume of a given multiplet glomerulus (one of multiple glomeruli in a given lateral or medial domain) [17]. Moreover, the sum of multiplet volumes in a given lateral or medial domain approximately equals the volume a singlet glomerulus. Thus, the size of each P2 glomerulus is inversely proportional to the number of P2 glomeruli occupying the same lateral or medial domain. As seen in figure 4*a*, singlet TAP^+/+^ glomeruli did not have significantly larger volumes than multiplet glomeruli. However, when we normalized each glomerular volume to the total glomerular volume in a given bulb, we did find significant differences between singlet and multiplet volumes. As shown in figure 4*b*, normalized volumes of singlet TAP^+/+^ glomeruli were approximately 50 per cent of the total glomerular volume in a given TAP^+/+^ bulb, whereas multiplet TAP^+/+^ glomeruli were approximately 25 per cent of the total P2 glomerular volume. These results match our previous findings that TAP^+/+^ bulbs average three glomeruli (two of which are multiplets and one of which is a singlet) and that total glomerular volume is evenly split between the lateral and medial domains. By contrast, TAP^−/−^ singlet glomeruli median volumes were significantly larger than their multiplet counterparts (figure 4*a*; Wilcoxon rank-sum test, *p* = 0.0020). Moreover, normalized volumes between singlets and multiplets were also significantly different: 0.44 ± 0.05 and 0.11 ± 0.02, respectively (figure 4*b*).

We found no significant difference in the total P2 glomerular volume between TAP^+/+^ and TAP^−/−^ bulbs (2.2 *±* 0.23 × 10^6^ versus 2.8 *±* 0.22 × 10^6^ μm^3^, respectively; Wilcoxon rank-sum test, *p* = 0.0769). We did, however, see a significant difference in the median normalized volume of TAP^−/−^ multiplet glomeruli when compared with TAP^+/+^ multiplet glomeruli (0.11 ± 0.02 versus 0.25 ± 0.05, respectively; Wilcoxon rank-sum test, *p* = 0.028). As the total P2 glomerular volumes did not differ between the two animal groups, the reduction in the median normalized volume for TAP^−/−^ multiplet glomeruli suggests that TAP^−/−^ animals had an increased number of smaller, P2 multiplet glomeruli. In fact, such an increase in smaller glomeruli can be seen in figure 4*c*,*d*. These results indicate that the increase in the number of multiplet glomeruli we find in the TAP^−/−^ bulbs results in smaller multiplet glomeruli when compared with the multiplet glomeruli seen in TAP^+/+^ bulbs.

### 3.5. P2 glomeruli are found in more variable locations in TAP^−/−^ main olfactory bulbs

**Figure 5.**
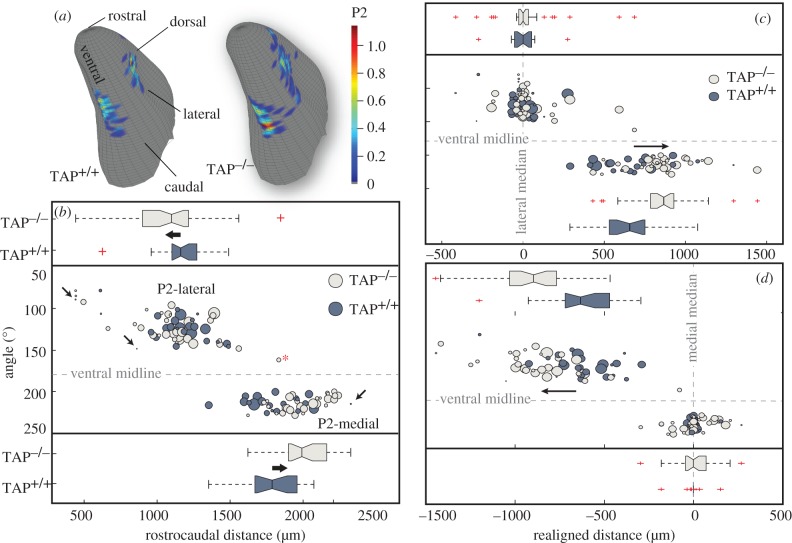
TAP^−/−^ animals have shifted P2 glomeruli along the rostrocaudal axis and enlarged olfactory bulbs. (*a*) Three-dimensional representations of the IGL from the MOB. Colour maps indicate the average density of X-gal positive glomerular cross sections from all bulbs in TAP^+/+^ or TAP^−/−^ animals. (*b*) Scatter plot. The cylindrical coordinates of collated P2 glomeruli from 14 TAP^+/+^ and 14 TAP^−/−^ MOBs. The diameter of each circle is proportional to the volume of the glomerulus. Arrows: minuscule TAP^−/−^ glomeruli. Asterisk: an exceptional TAP^−/−^ P2 outlier. Notched box plots*:* notches that do not overlap have medians that differ at the 5% significance level. Outliers that are more than 1.5 times the interquartile range are displayed with a red + sign. Rostrocaudal positions for TAP^+/+^ (blue) and TAP^−/−^ (beige) P2 glomeruli for P2-lateral glomeruli (top) and P2-medial glomeruli (bottom). Note the larger range of rostrocaudal positions for the TAP^−/−^ glomeruli. Large arrows highlight the significant shift in the median position towards the rostral end of the bulb (rank-sum test, *p* = 0.05). The bottom panel contains rostrocaudal plots for the P2-medial glomeruli. The right arrow highlights the significant shift in the median position of the P2-medial glomeruli towards the caudal end of the bulb (rank-sum test, *p* = 6.3 × 10^−4^). (*c*) Location of P2 glomeruli realigned to the median P2-lateral glomerular position in each bulb. Arrow highlights the 200+ μm caudal shift in the median position of TAP^−/−^ P2-medial glomeruli compared with their TAP^+/+^ counterparts (rank-sum rest, *p* = 0.0011). (*d*) Location of P2 glomeruli realigned to the median P2-medial glomerular position in each bulb. Arrow highlights the approximately 257 µm rostral shift in the location of TAP^−/−^ P2-lateral glomeruli when compared with their TAP^+/+^ counterparts (rank-sum test, *p* = 4.0010 × 10^−6^).

In MHCI-deficient mice, retinal afferents target a significantly expanded region of the LGN [1,33]. To characterize any positional variability of P2 glomeruli on the surface of MOBs in TAP^−/−^ animals, we mapped and plotted the location of each glomerular cross section that was positive for blue X-gal dye. The corrected, binned locations of all X-gal positive glomerular cross sections are shown in figure 5. In figure 5, two-dimensional histograms of P2 glomerular count are overlaid onto the surface of three-dimensional representations of the MOB. TAP^−/−^ histograms had positive values in more bins (113) than the corresponding TAP^+/+^ histograms (81; chi-squared test, *p* = 0.0187). These results suggest that more P2 glomeruli occupied a larger portion of the glomerular surface in TAP^−/−^ bulbs when compared with TAP^+/+^ bulbs.

In the scatterplot shown in figure 5*b*, each plotted circle represents the location of a single glomerulus, and the diameter of this circle is directly proportional to the calculated volume of the glomerulus. P2-lateral glomeruli fell rostrally between 441 and 1593 μm along the rostrocaudal axis and had angular coordinates ranging from 79° to 149°. P2-medial glomeruli fell more caudally along the rostrocaudal axis between 1353 and 2326 μm, and had angular coordinates ranging from 200° to 229°. While the two groups of glomeruli overlapped slightly along the rostrocaudal axis, we found no overlap in their angular coordinates. Of note, we found one TAP^−/−^ P2 glomerulus that fell well outside the P2-lateral or -medial domains (figure 5*b*, red asterisk). Owing to its exceptionally ectopic position, we excluded this outlier from our statistical comparisons of glomerular location.

We found no significant difference in the angular coordinates of P2 glomeruli between the TAP^+/+^ and TAP^−/−^ animals of either group of glomeruli. We calculated the average angle for TAP^+/+^ and TAP^−/−^ P2-lateral glomeruli to be 124.6 ± 12.7° and 124.5 ± 18.9°, respectively (rank-sum test, *p* = 0.7144). We calculated the average angle for P2-medial glomeruli TAP^+/+^ and TAP^−/−^ to be 214.4° and 212.7°, respectively (rank-sum test, *p* = 0.75). The angular coordinates for the P2 glomeruli in both domains are consistent with our previous findings for P2iTLZ glomeruli [17]. These results show that a reduction in the expression of MHCI molecules does not appear to affect the location of P2 glomeruli along the dorsoventral axis of the MOB.

In contrast to the angular coordinates, we found significant shifts in the location of P2 glomeruli along the rostrocaudal axis in TAP^−/−^ animals (figure 5*b*). We calculated the median rostrocaudal position for P2-lateral TAP^+/+^ glomeruli to be 1160.9 *±* 188.30 (±s.d.) μm—comparable with previous reports [34]. We calculated the median position for TAP^−/−^ P2-lateral glomeruli to be 1094.5 ± 296.5 µm, a 66.4 µm rostral shift along the rostrocaudal axis (rank-sum test, *p* = 0.05). For P2-medial glomeruli, we found an even greater but opposing caudal shift along the rostrocaudal axis. We calculated the median rostrocaudal position for P2-medial TAP^+/+^ and TAP^−/−^ glomeruli to be 1787.5 and 1991.5 µm, respectively—a shift of 204 μm (rank-sum test, *p* = 6.3 × 10^−4^). We confirmed this position shift in the P2-medial glomeruli by realigning the rostrocaudal origin for each MOB to the median rostrocaudal position for either the P2-lateral or P2-medial glomeruli in each MOB. In this manner, we established the lateral or medial median positions as fiducial markers independent of our mapping alignment (figure 5*c*,*d*). In both cases, we found commensurate increases in the separation between the TAP^+/+^ and TAP^−/−^ P2-lateral and P2-medial glomeruli. These results demonstrate that the loss of MHCI molecules appears to affect the position of P2 glomeruli in TAP^−/−^ animals along the rostrocaudal axis, culminating in a significant rostrocaudal separation between the P2-lateral and P2-medial glomeruli in TAP^−/−^ animals.

### 3.6. Olfactory bulbs in TAP^−/−^ mice are larger

**Figure 6.**
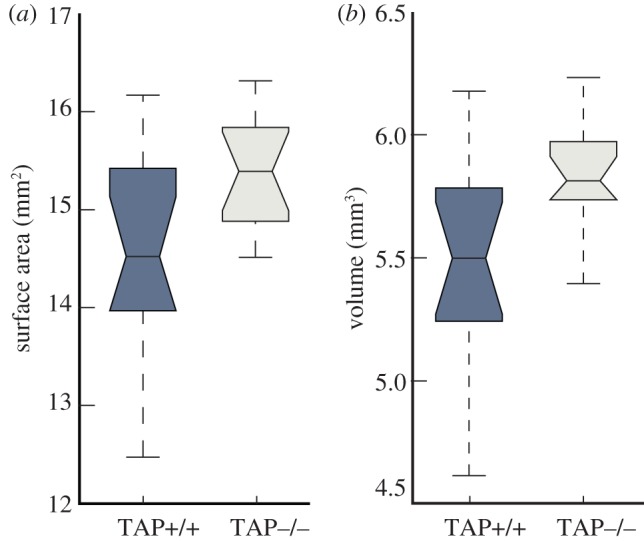
TAP^−/−^ bulbs have an increase calculated surface area and volume. (*a*) Estimated surface area of the inner glomerular layer (IGL) of olfactory bulbs from TAP^+/+^ (blue) and TAP^−/−^ (beige) animals. The calculated surface area is significantly larger in TAP^−/−^ bulbs (*t*-test, *p* = 0.01). (*b*) Calculated bulb volumes are significantly larger in TAP^−/−^ animals (*t*-test, *p* = 0.018).

One explanation for the average increased separation between the P2-lateral and P2-medial glomeruli would be an increase in the average overall size of olfactory bulbs in TAP^−/−^ animals, particularly along the rostrocaudal axis. In order to detect any such changes in the volumes of TAP^−/−^ bulbs, we calculated the surface area and volume of the inner glomerular layer (IGL) for all MOBs. As indicated by the non-overlapping notches in the box plots of figure 6*a*,*b*, both the surface area and volume of the IGL were significantly larger in the TAP^−/−^ animals than in the control littermates. We calculated the mean surface area for TAP^+/+^ and TAP^−/−^ IGLs to be 14.5 and 15.4 mm^2^, respectively (*t*-test, *p* = 0.01). We calculated the mean volume for TAP^+/+^ and TAP^−/−^ IGLs to be 5.50 and 5.82 mm^3^, respectively (*t*-test, *p* = 0.018). These results demonstrate that the surface area and volume of the IGL in the MOB are larger in TAP^−/−^ animals than in their littermate controls.

## 4. Discussion

In this study, we find that P2iTLZ TAP^−/−^ animals, which coexpress the tau-lacZ axonal marker with the P2 OR and do not express TAP, have a dramatic increase in the number of multiplet P2 glomeruli per MOB domain when compared with TAP^+/+^ animals. These duplicate P2 glomeruli were smaller on average than their TAP^+/+^ counterparts, and a subset of these glomeruli were morphologically abnormal. In addition, we find that the supernumerary P2 glomeruli occupied ectopic locations covering an expanded surface of TAP^−/−^ bulbs, especially along the rostrocaudal axis. This result is similar to the expanded targeting of retinal afferents to ectopic regions of LGN previously reported in an MHCI-compromised background [2]. Moreover, we find an increase in separation between the lateroventral P2 glomeruli and their medioventral counterparts in TAP^−/−^ animals, and a corresponding increase in the length of the rostrocaudal axis in TAP^−/−^ MOBs. Consistent with previous reports that MHCI-compromised brains appear grossly normal [2], we find no major anatomical abnormalities in TAP^−/−^ main olfactory tissue; careful examination of all TAP^−/−^ MOBs revealed grossly normal morphology, with all major cortical layers intact. Furthermore, we demonstrate that MHCI molecules are expressed in the main olfactory bulb (MOB) in adult C57Bl/6 animals and that the expression of these molecules is reduced in olfactory tissue of adult TAP^−/−^ mice. These results suggest that MHCI may be involved in the process that aids in coalescing broadly targeting P2 OSN to single glomeruli or in the mechanism that inhibits supernumerary glomeruli from persisting into adulthood.

Although the TAP^−/−^ mutation interferes with the presentation of only a subset of MHCI molecules on cells, our mutant phenotype is in line with recent data that show the disruption of just two MHCI genes (out of 50+) is sufficient to interfere with developmental refinement of retinogeniculate projections [35]. The increase that we find in the surface area and volume of the IGL in TAP^−/−^ MOBs is far larger than can be accounted by the slight increase in summed volume of TAP^−/−^ P2 glomeruli. This increase was confirmed by the increase in separation between TAP^−/−^ P2-lateral and P2-medial glomeruli. Such a significant rostrocaudal elongation of the MOB could potentially indicate that the targeted mutation of TAP resulted in a more widespread increase in supernumerary glomeruli than could be detected by examining the P2 glomerulus alone. Whereas the rostrocaudal position of the P2 glomerulus has been correlated to bulb size, the relative location of P2 glomeruli in uncompromised MHCI bulbs remains constant [30]. As the locations of the P2 glomeruli reported in this study have been realigned and scaled to a standard space, the relative position of P2 glomeruli in the TAP^−/−^ bulbs is clearly shifted relative to the position of glomeruli in TAP^+/+^ bulbs. This suggests that a mechanism other than a simple increase in MOB size could be responsible for the ectopic location of a subset of P2 glomeruli in this study. However, we cannot exclude the possibility that more subtle changes in the shape of the bulb, resultant from the TAP^−/−^ mutation, affected the location of the P2 glomeruli in this study.

### 4.1. Activity-dependent refinement of OSN axons into mature glomeruli

Similar to retinogeniculate projection targeting in the visual system [3,6,36], cAMP-reliant transcriptional regulation establishes axonal projection along the rostrocaudal axis of the MOB [18]. During development, activity-dependent processes ensure that each glomerulus is innervated from a homogeneous population of OSNs in the proper location on the MOB and that supernumerary glomeruli are (usually) eliminated [18,27–29,37]. However, this process appears highly plastic. For example, a recent study has shown that chronic exposure to novel odours not typically encountered by a mouse can lead to the formation of supernumerary glomeruli that persist into adulthood [38]. Moreover, disruption of activity in OSNs or in mitral cells can result in mistargeting of OSN projections to ectopic regions of the MOB and/or the formation of supernumerary glomeruli [29,32,39]. For example, sensory deprivation, such as naris occlusion, has been shown to result in the maintenance of multiple heterogeneous glomeruli [26,40] and olfactory learning has been shown to markedly accelerate glomerular maturation, resulting in early elimination of supernumerary glomeruli [41]. The phenotype that we report in this study is in line with a disruption in this very plastic, activity-dependent process of glomerular formation on the surface of the MOB.

### 4.2. The site of major histocompatibility class I molecule action

Owing to the diffuse and widespread nature of the pan-specific MHCI immunolabel throughout the adult olfactory tissue, we were unable to confirm exclusive colocalization with known immunomarkers of the different olfactory cell types typically found in the glomerular and nerve layers of the MOB. However, a recent study shows that an MHC I mRNA subtype is expressed throughout the glomerular and mitral cell layers of the MOB during perinatal time points well associated with glomerular formation and consolidation. Interestingly, no signal was seen for this particular mRNA subtype in the adult tissue. In the same study, MHCI protein was found to colabel with the neuronal marker NeuN in the calbindin-positive interneurons of the mitral cell and glomerular layers of P15 MOB bulbs [14]. Such an expression pattern would be in line with a post-synaptic role for MHCI molecules in olfactory circuits, as has been reported in other systems [7,42]. Nevertheless, the heterogeneity of these results highlights the complex and differential expression pattern of the 50+ MHCI genes in the mouse olfactory system and will require further detailed investigation to fully understand MHCI action in this system.

### 4.3. Conclusions

The stochastic nature of the mutant phenotype we describe in these TAP^−/−^ animals underscores the complex and heterogeneous mechanisms responsible for sorting, resolving and maintaining glomeruli on the surface of MOBs. To date, few global genetic or mechanical disruptions have successfully interfered with the formation of glomeruli in MOBs. Instead, regional disruptions affecting only a few glomeruli have proved illustrative and have highlighted the importance of competitive forces in the formation of glomeruli [32,39,43]. Moreover, previous studies have demonstrated a heterogeneous developmental regime for different glomeruli across the surface of the bulb [24,25,32,44]. Therefore, a careful characterization of other labelled glomeruli in the same and different MHC-compromised backgrounds in which the presentation of other subsets of MHCI molecules are affected warrants further consideration. The increase in supernumerary, ectopically located P2 glomeruli in an environment where the expression of a distinct subset of MHI molecules on cell surfaces is severely diminished serves as an additional, provocative piece to this very complex puzzle.

## 5. Material and methods

### 5.1. Animals

Dr Peter Mombaerts (Rockefeller University, New York, NY) kindly provided the P2-IRES-tau-lacZ (P2iTLZ) and P2-IRES-tauGFP (P2iGFP) mice, which coexpress the P2 OR with either the tau-lacZ or tauGFP fusion proteins in OSNs [23,45]. Tap1^tm1Arp^ mice severely deficient in the expression of MHCI molecules on cell surfaces were obtained from the Jackson Laboratory (Bar Harbor, ME). Tap1^tm1Arp^ animals were backcrossed over several generations to the background of P2iTLZ mice. We verified all genotypes by polymerase chain reaction. To control for sexual dimorphism, only anestrous female mice were compared. Mice were group-housed in conventional-style rodent cages containing separate water and food that could be obtained ad libitum. These cages contained HEPA filter cage tops that allowed air exchange through diffusion. Soiled litter was replaced every other week. All animals used were sacrificed according to institutional guidelines at 12 weeks of age, unless otherwise indicated.

### 5.2. Immunohistochemistry

Mice were anaesthetized with ketamine/xylazine (100 and 20 g/g body weight, respectively), and perfused transcardially with 0.1 M phosphate buffer (PB) followed by a phosphate-buffered fixative containing 3 per cent paraformaldehyde, 0.019 M l-lysine monohydrochloride and 0.23 per cent sodium m-periodate [46]. Bulbs were harvested and post-fixed for 2 h. Transverse sections (20 µm) were cut using a cryostat, mounted on Superfrost plus slides (Fisher Science, Pittsburgh, PA), and stored in a 80°C freezer until used. Sections or stripped epithelium were rinsed and incubated in blocking solution containing 2 per cent normal donkey serum, 0.3 per cent Triton X-100 and 1 per cent bovine serum albumin in PBS for 1.5 h. Sections were incubated for 72 h with primary antibodies against each of the following proteins: MHCI (1 : 100, T-2105, ER-HR52, BMA Biomedicals, Augst, Switzerland; specific for allotypes Kk, Dd, H-2q,r,s,v), GFAP (1 : 200; Z0334, Dako, Glostrup, Denmark), GAP-43 (1 : 200; 1752-S, Epitomics, Burlingame, CA; OMP, 1 : 200; 544-10001-wako, Wako, Richmond, VA) and p-75 (anti-nerve growth factor receptor, p75, 1 : 200; AB1554, Millipore, Billerica, MA). After incubation of the primary antibodies, sections were washed and reacted with secondary antibody (Alexa 488 or 568, Molecular Probes, Eugene, OR) for 1 h at room temperature. Sections were mounted on slides with Fluoromount-G. Controls for all immunohistochemistry experiments consisted of removing primary antibodies.

### 5.3. Whole mount X-Gal staining

We obtained the protocol and reagents from Specialty Media (subsidiary of Millipore, Billerica, MA). Fresh main olfactory tissue from sacrificed animals was immediately fixed for 30 min on ice with 0.1 M PB (pH 7.4), 4 per cent paraformaldehyde, 2 mM MgSO_4_ and 5 mM EGTA. The tissue was rinsed and washed for 5 min in tissue rinse solution A (0.1 M PB (pH 7.4), 2 mM MgCl_2_ and 5 mM EGTA), and then rinsed and washed for 30 min in tissue rinse solution B (0.1 M PB (pH 7.40), 2 mM MgCl_2_, 0.01% sodium desoxycholate and 0.02% Nonidet P-40). The tissue was then exposed for 2 h at 37°C in complete-gal tissue stain solution (40 mg ml^−1^ X-gal dissolved in DMSO) to generate the blue precipitate. The tissue was then cryosectioned, counterstained with 1 per cent neutral red, dehydrated with ethanol, cleared with xylene and mounted.

### 5.4. Imaging

All images were digitally captured using a Nikon (Tokyo, Japan) Eclipse E600 microscope equipped with epifluorescence and a Spot RT camera (3.04 magnification at the CCD chip).

### 5.5. Mapping glomeruli

We used the mapping technique detailed by Salcedo *et al*. [21]. Briefly, frozen, X-gal-stained bulbs were cryosectioned along the long axis of the bulb (L plane), perpendicular to the lateral olfactory tract, in serial sections 18 µm thick. We used our GLOM-MAP mapping software toolbox running in Matlab (The MathWorks, Natick, MA) to establish a coordinate axis on each digital image of sequential MOB tissue sections cut at 18 µm intervals (figure 2*a*). The rostrocaudal location of each tissue section was determined by the sequential position of the tissue on the glass slides. Unless otherwise indicated, distances along the rostrocaudal axis are measured from the caudal end of the bulb. Angular coordinates were established using internal anatomical landmarks for each tissue cross section so that the dorsal surface of the tissue corresponded to 0°, the lateral surface to 90°, ventral to 180° and medial to 270° (1). We segmented all glomeruli positive for the blue X-gal dye using a glomerular tracing tool in GLOM-MAP. The cylindrical coordinates of all glomerular cross sections were binned into 10 and 72 µm bins and then averaged across all bulbs in either the TAP^+/+^ or TAP^−/−^ bulbs to generate the density maps shown in figure 5*a*.

### 5.6. Glomerular reconstructions and standard bulb fittings

Individual glomeruli were digitally reconstructed from their component cross sections. The limits of a given glomerulus were determined by sequential tissue cross sections that did not contain X-gal dye at the corresponding cylindrical coordinates. The volume of each P2 glomerulus was calculated by summing its component cross-sectional areas and multiplying that sum by the section thickness (18 µm). The rostrocaudal position for each reconstructed glomerulus was set as the median rostrocaudal position of its component glomerular cross sections. In order to better align the cylindrical coordinates of P2 glomeruli between intra- and inter-animal MOBs, we aligned each bulb in three-dimensional space to a previously generated standard bulb. This standard bulb represents a digital reconstruction of the IGL (defined as the boundary between the glomerular layer and the external plexiform layer) from 16 adult C57BL/6 mouse MOBs [47]. The standard bulb consists of a collection of nodes, each with cylindrical coordinates *r*, *ϕ* and *z*. Steps for these nodes are 10° for *ϕ* and 18 µm for *z*, and have an associated distance within lattice to an optimal location. To fit to the standard bulb, we first segmented the IGL on each tissue cross section from all bulbs at 72 µm intervals using GLOM-MAP. Next, we used an algorithm from within GLOM-MAP to apply a multidimensional unconstrained nonlinear minimization (Nelder–Mead method) to find the position for all mapped points of the IGL from a given bulb that minimizes the mean distance of these points from the optimal position in the standard bulb. During the minimization process, GLOM-MAP preserves the mutual positions of the mapped point inside each section and mutual distances between sections. Finally, GLOM-MAP applies a bulb scaling factor to account for individual bulb size differences. For the purposes of this study, all of the MOBs from both strains of animals were fitted to the standard bulb, resulting in a re-calibration of the cylindrical coordinates for all glomeruli in these bulbs into the standard space. The volume and surface area for each MOB was similarly calculated with reference to the volume and standard area of the standard bulb. For each node of the standard bulb, volume was defined as *V* = 0.5 × *r*^2^ × *ϕ* × *z*. The area associated with each node was defined using Heron's formula, which can be stated as
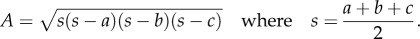
For the mapped bulbs in the TAP^+/+^ and TAP^−/−^ animals, volume and surface area were defined as sums of node-volumes and node-areas for all nodes in the close proximity to the mapped perimeter. In the regions marked as damaged tissue during the segmentation of the IGL, volume and surface area were extrapolated to the closest nodes in the standard bulb. To standardize comparisons across animals, all IGL volume and surface area calculations were cropped at the caudal end of the bulb to the minimum mapped axis length of 3178.4 µm.

### 5.7. Statistics

We used the Statistics Toolbox for Matlab for the majority of statistics presented here, such as the two-tailed Student's *t*-test, Wilcoxon rank-sum test and Pearson's chi-squared test. We also used Matlab to generate all of the scatterplots, cumulative distribution plot, histograms and box plots. For the notched box plots, the width of the notches was computed, so that box plots whose notches do not overlap had different medians at the 5 per cent significance level, assuming a normal distribution [48]. The left and right side of each box are the 25th and 75th percentiles, respectively. The centre black line in each box plot is the median value. Outliers that are more than 1.5 times the interquartile range are displayed with a red + sign. Where *t*-tests where reported, we tested for a normal distribution in the samples by using Levene's test at the 5 per cent significance level. To determine significance for the number of glomeruli per bulb, we performed a mixed-effects analysis ANOVA using the SAS procedure MIXED (SAS, Cary, NC). In this procedure, group (TAP^+/+^ or TAP^−/−^), bulb handedness (left or right) and location were considered fixed effects. Mice were considered random effects nested within each group combination. Side and location were treated as repeated-measure factors on each glomerulus.

## 6. Acknowledgements

This work was supported by grants from the National Institute on Deafness and Other Communication Disorders for D.R. and E.S. This work is also supported by the Rocky Mountain Taste and Smell Center.
